# Editorial: Wearable Sensors for Remote Health Monitoring and Intelligent Disease Management

**DOI:** 10.3389/fspor.2021.788165

**Published:** 2021-12-01

**Authors:** Emma Fortune, Jeremy R. Crenshaw, Jacob J. Sosnoff

**Affiliations:** ^1^Robert and Patricia Kern Center for the Science of Health Care Delivery, Division of Health Care Delivery Research, Mayo Clinic, Rochester, MN, United States; ^2^Falls and Mobility Laboratory, Department of Kinesiology and Applied Physiology, University of Delaware, Newark, DE, United States; ^3^Department of Physical Therapy, Rehabilitation Science, and Athletic Training, University of Kansas Medical Center, Kansas City, KS, United States

**Keywords:** wearable sensors, remote health monitoring, accelerometers, inertial measurement unit, electrocardiography, photoplethysmography, physical activity, heart rate variability

Aging populations are increasing at an unprecedented rate, leading to increased numbers of people requiring medical attention and frequent monitoring. This will pose major challenges to health and social systems and will result in a significant socio-economic burden (Harper, 2014). Remote health monitoring can allow for low-cost health outcomes to be tracked for preventative measures and disease management. This will enable people to live longer at home, reduce the need for clinic visits as well as reduce provider burden, and aid with clinical decision-making using real-world objective health outcomes. This is a rapidly expanding field, with end-user spending on wearable devices totaling $81.5 billion worldwide in 2021 with a significant recent increase in market growth due to increased remote working and health monitoring.

In November 2019, we launched the Research Topic “Wearable Sensors for Remote Health Monitoring and Intelligent Disease Management” in *Frontiers in Sports and Active Living* with the following aims:

(i) to develop novel utilizations of wearables to assess health outcomes in a range of populations;
(ii) to evaluate promising wearable technology and/or data analysis approaches designed to monitor health outcomes;
(iii) to reveal directions for future development.

This Research Topic includes eight articles by 41 authors and has received high interest with more than 21,000 views as of September 2021. We summarize the key features of this collection below (Table 1).

**Table 1:** Summary of all studies within the Research Topic including participants involved, devices used, data collection type, software employed, sensor-based variables, and primary outcome.

**Reference**	**Participants**	**Devices**	**Data collection type**	**Software employed**	**Sensor-based variables**	**Primary outcome**
Chopra et al.	70 postmenopausal women [mean (minimum–maximum) age 61 (46–79) years] with range of bone mineral densities	Actigraph accelerometer on the ankle	Field-based measurement for 7 days	Custom algorithms in Matlab to analyze raw data	Active time, sedentary time, sedentary break (number, distribution, time accumulation pattern, length variability) step count, stepping bout (distribution, time accumulation pattern, length variability), sedentary bout (length, distribution, time accumulation pattern)	Healthier hip bone mineral density may be associated with PA distributed more evenly throughout the day with shorter sedentary bouts. PA distribution should be considered, in exercise-based bone health management programs
Frechette et al.	11 wheelchair users [mean (SD) age: 35 (18) years]	Smartphone accelerometer on chest	Lab-based validation	Custom algorithms in Matlab to analyze raw data	Postural control: maximum accelerations along the mediolateral and anteroposterior axes	Validity and reliability of smartphone accelerometry was comparable to the research-grade accelerometer and clinical tests of postural control, suggesting its ability to assess seated postural control and distinguish between those with and without impaired postural control
Andreasen et al.	142 stroke survivors [mean (SD) age: 63 (11) years]	Fitbit on the ankle	Field-based measurement for 7 days to test associations with weather	Fitbit; custom algorithms in Matlab to analyze step counts per minute data from Fitbit	Step count, gait non-linear structure and complexity	Stroke survivors with high step counts are active at similar times each day and have higher activity complexities as measured through patterns of movement at different intensity levels. Weather affects our activity parameters in terms of complexity between spring and winter.
Kuntapun et al.	12 young [mean (SD) age: 23 (2) years] and 12 old adults [mean (SD) age: 76 (6) years]	Smartphone accelerometer with smartphones placed in a shoulder bag/on the lumbar spine	Lab and field based validation	Custom algorithms in Matlab to analyze raw data	Gait velocity, step time, step length, cadence, center of mass	Results showed that smartphones were reliable and valid for measuring gait across all conditions, phone placements, and environments. Lumbar spine placement of the smartphone was reliable and valid for center of mass measurement
						Smartphones demonstrate the potential for remote patient monitoring and home health care
Youn et al.	18 older adults [mean (SD) age: 67 (8) years] at least 6 months after total knee arthroplasty	Ankle-worn accelerometers (Moraxon)	Lab-based association testing	Custom algorithms in Matlab to analyze raw data	Step vector magnitudes (whole and initial 10%), heel-strike magnitude (Iateral, vertical, and anterior), heel-strike impulse (Iateral, vertical, and anterior), stance phase angle variation (lateral, anterior), step time	Inertial gait variables were significantly correlated with performance test and questionnaire outcomes but did not correlate well with isometric strength measures. Wearable sensor-based gait analysis may help predict clinical measures in individuals after unilateral knee treatment.
Baroudi et al.	10 participants (minimum–maximum age: 22–38 years)	Thigh worn activPAL	Lab-based validation and field-based measurement in one participant (7 days)	Custom algorithms in Matlab to analyze raw data	Walking speed	The approach presented here provides promising results that can enable clinicians to complement their existing assessments of activity level and fitness with measurements of movement duration and intensity (walking speed) extracted at a week time scale and in the patients' free-living environment.
Stone et al.	5 healthy young adults [mean (SD) age: 20 (2) years]	Shimmer3 mECG unit, Polar H10, Firstbeat textile strap, OURA ring, iphone 8	Lab-based validation	Commercial software application	Heart rate and heart rate variability	Study results demonstrated varying degrees of accuracy among the different devices. To thoroughly address questionable claims from manufacturers, elucidate the accuracy of data parameters, and maximize the real-world applicative value of emerging devices, future research must continually evaluate COTS devices.
Goodwin et al.	44 MWC users and 44 controls [mean (SD) age 43 (12) years]	Arm and chest inertial measurement units	Field-based measurement	Custom algorithms in Matlab to analyze raw data	Percentage of the day and daily minutes spent static and dynamic at different humeral elevation ranges.	MWC users spent more time dynamic in higher elevations than controls, and with age, dynamic arm use decreased in the 30–60° humeral elevation range. These results may exemplify effects of performing activities from a seated position and of age on mobility.

## Utilizing Smartphones as Wearables

According to Pews Research Center, 85% of U.S. adults own a smartphone. This presents a ripe opportunity for remote health monitoring providing the data are sufficiently accurate. Kuntapun et al. demonstrated that smartphones are reliable and valid for measuring gait variables (walking speed, step length, step time, and cadence) in young and old able-bodied adults either when placed in a shoulder bag or attached to the lumbar spine, and for measuring center of mass displacement with lumbar spine placement. Frechette et al. validated the use of a chest-affixed smartphone to measure postural control in wheelchair users during standard clinical tests and pilot-tested its ability to identify those with impaired postural control, indicating the potential of conducting assessments remotely in a patient's home environment. Stone et al. tested the validity of an iPhone camera paired with commercially available smartphone applications to measure heart rate and heart rate variability in young healthy adults.

## Research- and Consumer-Grade Wearables

Some studies utilized consumer-grade wearables such as a FitBit (Andreasen et al.) to estimate their outcome variables. However, many still prefer to develop and/or apply their own custom algorithms to the raw data to allow for increased flexibility and complexity of data analyses, as well as algorithm transparency. This also allows for algorithms to be easily translated for use across different devices with similar specifications. Stone et al. investigated the validities of commercial devices and associated platforms to measure heart rate with results of varying accuracies. These findings reinforce the importance of evaluating device validity before use in real-world applications, especially as most commercial devices have proprietary steps in their data analyses. This is particularly needed for special populations as increased measurement errors have frequently been reported for individuals with decreased or altered physical function (Dijkstra et al., 2008).

## Acceleration-Derived Health Outcomes

Most studies in this collection utilized acceleration data to derive their outcome measures and demonstrated the wide range of potential use of accelerometer-based sensors. Variables such as sedentary behavior, physical activity (active time, step counts, and walking speed), physical function, postural control, and balance can be assessed to determine health status and upper and lower body function in both young and old adults whether they are able-bodied, manual wheelchair users, or those of limited physical function status.

## Concluding Remarks

This Topic's articles have enhanced our knowledge on the capabilities of wearables to measure physical function and heart rate in adults of all ages during their daily lives. Novel uses of existing technologies have been evaluated and new approaches to analyzing physical function in daily life have been presented. However, much work is still needed for the effective usage of wearables for remote health management.

While some investigations involve the use of wearables in the real-world setting, the majority focus on validating existing and new movement parameters, particularly in the laboratory setting. Increasing evidence demonstrates mobility differences between laboratory and real-world settings (Warmerdam et al., 2020). However, further investigations into these differences and their implications in terms of both validation and utility in evaluations of deficits will be key for successful translation from research to practice. Therefore, we encourage future studies on validation and application of wearables in both settings and their ability to assess and predict clinical health outcomes. For remote monitoring to be effectively useful for disease management through early prevention and identification strategies as well as the reduction of healthcare costs, clinic visits, and hospitalizations, the integration of remote monitoring into clinical standards of care is of critical importance and by no means a small challenge.

The study cohorts investigated in this collection ranged from young (Kuntapun et al.; Baroudi et al.; Stone et al.) to older adults and included manual wheelchair users (Frechette et al.; Goodwin et al.), stroke survivors (Andreasen et al.), total knee arthroplasty patients (Youn et al.), and women with low bone mineral density (Chopra et al.). It is crucial that future research continues to expand on including a wide range of populations to address health disparities, particularly those resulting from racial/ethnic, sex and gender, disability, and socio-economic-based differences. Another area of vital importance that needs additional work is the usability of wearables for target populations.

From the work presented here, it remains uncertain whether these wearable devices can be used as preventative or interventional tools to improve or maintain the long-term physical health of aging adults. However, the use of wearables with feedback has been shown to elicit improvements in physical activity and function at least in the short-term (Brickwood et al., 2019). With the rapidly expanding improvements in wearable technology, especially with smartwatches and ear-worn devices, leading to increased long-term daily usage, the potential to include lifelong objective physical activity profiles will significantly increase the power of these devices to predict adverse health events and outcomes for individuals. Interest in wearables and remote monitoring will continue to grow, as will sensor innovations, accuracies, and miniaturization. We are excited to see how future novel developments and applications in this field will transform healthcare.

## Author Contributions

EF, JRC, and JJS wrote and edited the manuscript. All authors contributed to the article and approved the submitted version.

## Funding

This publication was supported by the National Institute of Aging and the National Center for Advancing Translational Sciences of the National Institutes of Health (NIH) under Grant Numbers U54 AG44170, K12 HD065987, and UL1 TR002377, in addition to the Mayo Clinic Robert D. and Patricia E. Kern Center for the Science of Health Care Delivery.

## Conflict of Interest

The authors declare that the research was conducted in the absence of any commercial or financial relationships that could be construed as a potential conflict of interest.

## Publisher's Note

All claims expressed in this article are solely those of the authors and do not necessarily represent those of their affiliated organizations, or those of the publisher, the editors and the reviewers. Any product that may be evaluated in this article, or claim that may be made by its manufacturer, is not guaranteed or endorsed by the publisher.
